# Advances in Anti-Tumor Treatments Targeting the CD47/SIRPα Axis

**DOI:** 10.3389/fimmu.2020.00018

**Published:** 2020-01-28

**Authors:** Wenting Zhang, Qinghua Huang, Weiwei Xiao, Yue Zhao, Jiang Pi, Huan Xu, Hongxia Zhao, Junfa Xu, Colin E. Evans, Hua Jin

**Affiliations:** ^1^Guangdong Provincial Key Laboratory of Medical Molecular Diagnostics, The Scientific Research Center of Dongguan, College of Pharmacy, Institute of Clinical Laboratory Medicine, Guangdong Medical University, Dongguan, China; ^2^Marine Medical Research Institute of Guangdong Zhanjiang, Zhanjiang, China; ^3^Biosafety Level-3 Laboratory, Guangdong Provincial Key Laboratory of Tropical Disease Research, School of Public Health, Southern Medical University, Guangzhou, China; ^4^Key Laboratory for Tropical Diseases Control of the Ministry of Education, Department of Microbiology, Zhongshan School of Medicine, Sun Yat-sen University, Guangzhou, China; ^5^School of Biomedical and Pharmaceutical Science, Guangdong University of Technology, Guangzhou, China; ^6^Feinberg School of Medicine, Northwestern University, Chicago, IL, United States

**Keywords:** CD47, CD47/SIRPa axis, immunotherapy, phagocytosis, signal-regulatory protein α (SIRPα)

## Abstract

CD47 is an immunoglobulin that is overexpressed on the surface of many types of cancer cells. CD47 forms a signaling complex with signal-regulatory protein α (SIRPα), enabling the escape of these cancer cells from macrophage-mediated phagocytosis. In recent years, CD47 has been shown to be highly expressed by various types of solid tumors and to be associated with poor patient prognosis in various types of cancer. A growing number of studies have since demonstrated that inhibiting the CD47-SIRPα signaling pathway promotes the adaptive immune response and enhances the phagocytosis of tumor cells by macrophages. Improved understanding in this field of research could lead to the development of novel and effective anti-tumor treatments that act through the inhibition of CD47 signaling in cancer cells. In this review, we describe the structure and function of CD47, provide an overview of studies that have aimed to inhibit CD47-dependent avoidance of macrophage-mediated phagocytosis by tumor cells, and assess the potential and challenges for targeting the CD47-SIRPα signaling pathway in anti-cancer therapy.

## Introduction

Despite rapid improvements in anti-cancer therapies, the incidence of cancer and the rates of morbidity and mortality in cancer patients remain unacceptably high. In 1957, Burnet and Thomas proposed the “immuno-monitoring” hypothesis, suggesting that the immune system can monitor and eliminate “exotic” entities to maintain a homeostatic environment, including malignant cells that express various tumor-specific as well as non-tumor-specific antigens (1). Tumor immunosurveillance is an important process of limiting tumor growth and macrophages play a major role in the recognition and removal of tumor cells from the body (2). T-cells and natural killer (NK) cells are other important effector cells of the immune system, which also play important roles in anti-tumor immunity (2). The evasive behavior of tumor cells from immune recognition and clearance depends on a multitude of processes and includes induction of an immunosuppressive tumor microenvironment and reductions in tumor cell immunogenicity (3, 4). One key mechanism of tumor cell immune escape is through overexpression of the immunosuppressive signaling molecule, CD47 (5–7). Widely regarded as a “don't eat me” signal, CD47 helps maintain immunotolerance by non-malignant cells under physiological conditions (7), but this same molecule can aid in the survival of cancer cells in various cancer types (5, 6). In many cancer types, CD47 binding to signal-regulatory protein α (SIRPα) initiates an inhibitory signaling pathway that leads to the evasion of malignant cells from phagocytosis by macrophages (8).

Immunotherapy aims to reduce tumor propagation through activation or modulation of the innate or adaptive immune systems (9). To improve understanding of the mechanisms used by immune cells to detect and eliminate cancer cells, several molecules and signaling pathways have been investigated (10). Given that the binding of CD47 with SIRPα in tumor cells limits the anti-cancer immune response, it is possible that therapies that inhibit CD47 signaling in cancer cells would promote the phagocytosis of tumor cells by macrophages and thereby limit tumor growth (11). For example, immune cells or antibodies that inhibit tumor cell-expressed CD47 could be used to reduce the growth and spread of tumors with high expression of CD47, providing a feasible immunological target for anti-tumor therapies (12). However, the CD47-mediated regulation of phagocytotic removal of different types of cancer cells remains incompletely understood. The aims of this review are to: (i) describe the structure and function of CD47; (ii) provide an overview of studies that attempt to promote macrophage-mediated tumor cell phagocytosis through antagonism of CD47 signaling; and (iii) discuss the potential and challenges for targeting CD47-SIRPα signaling in anti-cancer therapies.

### Characterization of CD47 Structure and Ligands

CD47 is a transmembrane protein that is glycosylated on the surface of a variety of different cell types (13, 14). Lindberg et al. isolated and purified CD47, which is also known as integrin-associated protein (IAP) (13). CD47 belongs to the immunoglobulin superfamily and is a supramolecular complex composed of integrins, G protein, and cholesterol (15). The structure of CD47 includes an amino terminal extracellular variable region, a transmembrane region formed of highly hydrophobic transmembrane segment, and a hydrophilic carboxy-terminal cytoplasmic tail that interacts with the corresponding ligands (15) to mediate a series of processes such as cell proliferation, migration, phagocytosis, and apoptosis, as well as immune homeostasis and inhibition of NO signaling (16, 17).

**Figure 1:**
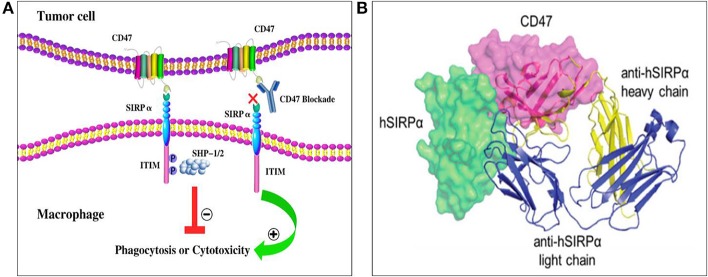
Structure and interaction of CD47 and SIRPα. **(A)** CD47 contains one N-terminal extracellular IgV-like domain and five transmembrane spanning segments. SIRPα contains three extracellular IgSF domains, one transmembrane spanning region, and an intracellular domain with ITIM motifs. After the binding of CD47 to SIRPα, two ITIMs in the cytoplasmic tail of SIRPα become phosphorylated then recruit and activate phosphatases including SHP-1 and SHP-2. Ultimately, CD47-SIRPα binding inhibits the host cell from being targeted for phagocytosis, while anti-CD47 antibodies can block the suppression signal and promote phagocytosis (18). **(B)** The anti-SIRPα antibody, KWAR23 (depicted as ribbons), binds SIRPα at an epitope overlapping with the CD47/SIRPα interface (19). Abbreviations: CD47, cluster of differentiation 47; ITIM, immunoreceptor tyrosine-based inhibitory motif; SHPS-1/2, protein tyrosine phosphatase substrate-1/2; SIRPα, signal-regulatory protein α.

The ligands of CD47 include SIRPα, thrombospondin-1 (TSP-1), and integrins including αvβ3 and α2β1 (15). SIRPα, also known as SHPS-1, is a transmembrane protein with an extracellular region containing three immunoglobulin superfamily-like regions: one NH2-terminal V-like structure domain and two C1-like IgSF domains, with the NH2 terminal domain able to bind to CD47 (Figure 1A). Examination of the CD47/SIRPα complex revealed that the anti-human SIRPα antibody, KWAR23, binds SIRPα at an epitope overlapping with the CD47/SIRPα interface, indicating a basis for competitive antagonism of the CD47/SIRPα interaction (Figure 1B). SIRPα is highly expressed on the membrane of myeloid cells, such as macrophages, granulocytes, monocytes, and myeloid dendritic cells (20). It regulates cell migration and phagocytic activity as well as immune homeostasis and neural network formation (16, 21). TSP-1 is a homotrimeric multi-domain extracellular matrix glycoprotein belonging to a family of extracellular secreted proteins that consist of a variety of domains known to bind extracellular matrix components and cell surface receptors (12). TSP-1 is secreted by platelets, monocytes, macrophages, and a variety of other non-hematopoietic cell types (22). TSP-1 binding to CD47 leads to changes in the concentration of intracellular calcium ions and cyclic adenosine/cyclophosphinoside that regulate cell survival and migration, and TSP-1 also induces cellular responses to tissue damage (12, 17).

### Pathophysiological Function of CD47

The expression of CD47 is used by macrophages to distinguish between “self” or “non-self” (7). CD47 is expressed on the surface of non-malignant cells as well as multiple types of cancer cells and can bind to the SIRPα transmembrane protein on myeloid cells (especially macrophages) to form the CD47-SIRPα signaling complex (23). The extracellular IgV domain of SIRPα binds to CD47, leading to tyrosine phosphorylation on the intracellular ITIM motif; SIRPα also binds to the SH2 domain-containing tyrosine phosphatases, both of which inhibit the accumulation of myosin IIA in phagocytic synapses and facilitate the release of “don't eat me” signals that inhibit macrophage-mediated phagocytosis and protect normal cells from being damaged by the immune system (8, 23). Conversely, when the surface expression of CD47 is reduced, for example in aged cells, the CD47-SIRPα signaling pathway is weakened (24, 25), and macrophages can move toward and phagocytose these cells (26). CD47 on normal erythrocytes binds to SIRPα on the surface of macrophages, producing an inhibitory signal that prevents phagocytosis (27), but when erythrocytes undergo senescence, the expression level of CD47 is decreased, and CD47-deficient senescent erythrocytes are regarded as foreign and rapidly cleared by macrophages in the spleen (27). Ishikawa-Sekigami et al. transferred normal erythrocytes into mice lacking intracellular ITIM motifs and found that these erythrocytes were rapidly phagocytosed, confirming that the CD47-SIRPα signaling pathway plays a major role in the phagocytosis of erythrocytes (28). CD47 and its ligands not only regulate the immune response, but also mediate various pathophysiological processes such as neutrophil chemotaxis and nervous system development, as well as playing a regulatory role in immune tolerance and T-cell activation (11).

### CD47 Expression in Cancer Cells and Tumors

Cancer cells exploit the “don't eat me” function of CD47 by expressing higher levels of CD47 on their surface compared with non-malignant cells; numerous studies have shown that CD47 is overexpressed in different types of tumors (16), including in myeloma (29), leiomyosarcoma (30), acute lymphocytic leukemia (31), non-Hodgkin's lymphoma (32), breast cancer (33), osteosarcoma (34), and head and neck squamous cell carcinoma (23). For example, studies by Russ et al. demonstrated that CD47 is overexpressed in acute myeloid leukemia (AML) and chronic myeloid leukemia (CML) cells when compared with normal myeloid cells from healthy mice or humans; these authors also showed that higher CD47 expression levels were associated with worsened therapeutic response and prognosis (35). Under inflammatory conditions and during cytokine mobilization, the expression of CD47 in normal hematopoietic stem cells is upregulated (36), and leukemia progenitor cells use this mechanism to evade macrophage-mediated phagocytosis (37). Majeti et al. (38) and Yang et al. (39) demonstrated that CD47 mRNA and protein levels in leukemia stem cells from AML patients were higher than those in normal healthy stem cells and that these increases were associated with poor prognosis. Galli et al. (40) found that CD47 is highly expressed in 25% of primary AML samples and that the expression of CD47 is inversely related to treatment response and favorable prognosis. Mohanty et al. found that the expression of CD47 in human osteosarcoma samples was higher than that of normal bone samples (34). Weiskopf et al. found that small cell lung cancer (SCLC) cell lines (NCI-H1688, NCI-H128, NCI-H524, NCI-H82, NCI-H69, and NCI-H196) expressed high levels of CD47 mRNA and protein (41). Compared to both normal peripheral blood and germinal center B cells, CD47 is more highly expressed on a large subset of primary patient samples from multiple B cell non-Hodgkin lymphoma (NHL) subtypes, including diffuse large B cell lymphoma (DLBCL), B cell chronic lymphocytic leukemia (B-CLL), mantle cell lymphoma (MCL), follicular lymphoma (FL), marginal zone lymphoma (MZL), and pre-B acute lymphoblastic leukemia (pre-B ALL), while high expression of CD47 is predictive of poor prognosis in NHL patients (42). CD47 is also overexpressed on myeloma cells, and its expression increases with the progression of multiple melanoma (MM) (43). Rendtlew Danielsen et al. (44) analyzed the expression of CD47 and TSP-1 and TSP-2 in plasmocytes from MM patients using fluorescence-activated cell sorting and found that CD47 and TSP-1 and TSP-2 ligands are highly up-regulated in the MM interstitial environment. Furthermore, CD47 is overexpressed on the surface of hepatocellular carcinoma (HCC) cells, and high expression of CD47 is associated with enhanced metastasis of liver cancer cells in transplanted mice (45). In a study of CD47 expression in four cholangiocarcinoma (CCA) cell lines (KKU-100, KKU-055, KKU-213, and HuCCT1) and 54 CCA tissues samples, high expression of CD47 was found in 3 out of 4 of the CCAs cell lines (KKU-055, KKU-213, and HuCCT1) and in 50 out of 54 of the CCA tissue samples (46). Wang et al. (47) used immunohistochemistry to detect CD47 in ovarian clear cell carcinoma tissues and showed that the survival rate of patients with low CD47 is higher than that of high-level patients, while CD47 level is predictive of the patient's disease stage, chemotherapy resistance, and prognosis. Li et al. (48) demonstrated that ovarian cancer patients with low expression of CD47 in their tumors have a better therapeutic response to standard treatment and overall survival compared with the patients with higher CD47 expression. Brightwell et al. (49) used the cancer genome atlas to test the association between CD47 expression and clinical outcome in epithelial ovarian cancer patients; immunohistochemical analysis of 265 patient specimens showed that CD47 expression was found in 210 of 265 (79%) patients, and that of these patients, high levels of CD47 protein expression was found in 129 of 265 (49%) patients. Nagahara et al. (50) evaluated CD47 and SIRPα mRNA levels in the bone marrow and peripheral blood of 738 breast cancer patients; these authors showed the survival rate of patients with high CD47 expression in their tumors was significantly lower than those with low CD47 expression levels. CD47 protein and mRNA levels are also overexpressed on the surfaces of lung cancer cells, and by SCLC tumors and non-small cell lung cancer (NSCLC) tumors (51). It has also been reported that CD47 is expressed on more than 80% of the surface of bladder cancer cells, and that the expression of CD47 in muscular invasive bladder cancer (MIBC) and non-muscle invasive bladder cancer (NMIBC) is significantly higher than that in normal urothelial cells (52). Li et al. (53) found that CD47 is highly expressed on glioma cells and glioma stem cells, while the levels of CD47 in 5 distinct patient-derived pancreatic ductal adenocarcinoma (PDAC) cell lines (T366, T395, T449, T608, and T738) are variable (54). In studies of Epstein-Barr virus-associated gastric carcinoma (EBVaGC), CD47 expression was increased in EBVaGC tissue samples compared to EBV-negative gastric cancer tissue samples (55). High expression of CD47 is also associated with poor prognosis in EBVaGC (55). Yoshida et al. (56) showed that gastric cancer cells with high CD47 expression show higher proliferation and spheroid colony formation than those expressing low levels of CD47. Conversely, another study indicated that there is no difference in the expression of CD47 mRNA between primary gastric cancer and normal gastric tissue (57). Furthermore, increased expression of CD47 mRNA is not an unfavorable prognostic factor in primary gastric tumors, suggesting that there may be post-transcriptional differences leading to increased expression of CD47 protein and that the reliance of disease progression upon CD47 expression could vary between different cancer types and patient populations. Future studies should continue to attempt to elucidate the respective role(s) of CD47 in different cancer types including pancreatic and gastric cancer (57).

## Mechanisms of Action and Impact of Targeting CD47-SIRPα Signaling in Anti-Tumor Therapy

**Figure 2:**
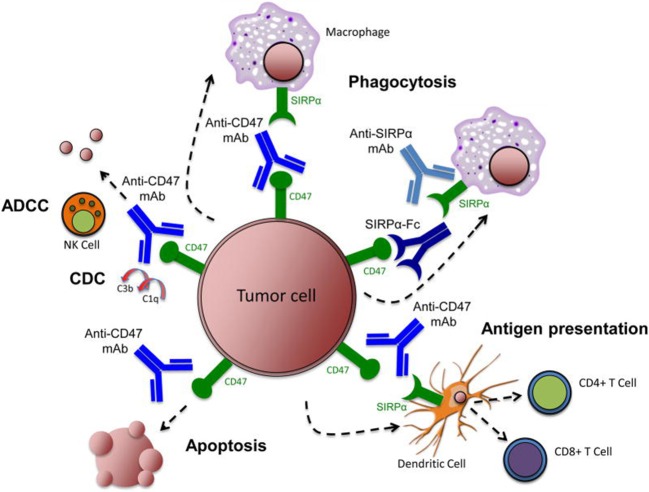
Targeting the CD47-SIRPα pathway in cancer. Therapeutic targeting of the CD47-SIRPα pathway can cause elimination of cancer cells through multiple mechanisms. First, inhibition of the CD47-SIRPα interaction using an anti-CD47 antibody, an anti-SIRPα antibody, or a recombinant SIRPα protein, leads to phagocytic uptake of tumor cells by macrophages. Second, anti-CD47 antibodies enable phagocytic uptake of tumor cells by dendritic cells and subsequent antigen presentation to CD4^+^ and CD8^+^ T-cells, thereby stimulating an anti-tumor adaptive immune response. Third, anti-CD47 antibodies eliminate tumor cells through natural killer cell-mediated antibody-dependent cytotoxicity and complement dependent cytotoxicity. Fourth, anti-CD47 antibodies stimulate apoptosis of tumor cells through a caspase-independent mechanism. Image reproduced with permission from Chao et al. (58). ADCC, antibody-dependent cell-mediated cytotoxicity; CDC, complement dependent cytotoxicity; mAb, monoclonal antibody; NK, natural killer; SIRPα, signal-regulatory protein α.

Overexpression of CD47 on the tumor cell surface can help these cells escape monitoring and clearance by immune cells, making CD47 a plausible target in the development of novel anti-tumor drugs (18). For example, anti-CD47 treatments have been used to block CD47-SIRPα inhibitory signaling and promote the phagocytosis of tumor cells by macrophages, but anti-CD47 treatments can exert their anti-tumor impact through a number of different mechanisms (Figure 2) (58).

### Impact of CD47/SIRPα Targeting on Macrophage-Mediated Phagocytosis

CD47 inhibition leads to stimulation of phagocytosis of cancer cells by macrophages (38, 41). Anti-CD47 antibodies enhance the phagocytosis of tumor cells by macrophages by directly blocking the binding of tumor cell-expressing CD47 to macrophage-expressing SIRPα (18). Treatment of mice with a macrophage-depleting chemical (clodronate) leads to abrogation of the anti-tumorigenic effects of CD47 blockade (38), supporting the role of macrophages in anti-CD47-mediated anti-tumor response. Zeng et al. (59) showed that the humanized anti-CD47 antibody, ZF1, blocks the interaction between CD47 and SIRPα, and enhances the phagocytosis of leukemia stem cells by macrophages. Through direct competition with SIRPα on the surface of macrophages, *E. coli*-constructed recombinant hSIRPext binds to CD47 on the surface of leukemia stem cells, which blocks CD47-SIRPα signaling (60). In their study, Lin et al. (60) combined the plasmid vector, pET32a, with the soluble extracellular domain of SIRPα, and imported the resulting construct into the *E. coli* strain, BL21, to obtain a CD47 fusion protein. Alternatively, they obtained another variant of the CD47 fusion protein by splicing the extracellular domain of human CD47 into a pET32a plasmid vector and importing this into the *E. coli* strain, BL21. Lin et al. (60) then co-incubated the 2 CD47 fusion proteins (Trx-hCD47ext and Trx-CD47ext) with Jurkat cells and showed that both the proteins enhance the phagocytosis of leukemia cells by macrophages *in vitro*. Other studies have shown that anti-CD47 antibodies promote macrophage phagocytic activity in human NHL cell-engrafted mice (61). Xu et al. (61) treated NHL mice with an anti-CD47 antibody and blinatumomab (which targets CD19 and CD3); this combination of therapies led to persistent control of lymphoma progression by inducing cancer cell phagocytosis and T-cell cytotoxicity. Hu5F9-G4 is a humanized IgG4 isotype monoclonal antibody against CD47 and a macrophage immunological checkpoint inhibitor that blocks CD47 to induce phagocytosis; previous studies have found that Hu5F9-G4 may act synergistically with rituximab to eliminate B-cell NHL cells by enhancing macrophage-mediated antibody-dependent phagocytosis (62). Weiskopf et al. (63) demonstrated that the anti-tumor synergistic impact of Hu5F9-G4 and rituximab treatment depends largely on macrophage-mediated tumor killing in a canine lymphoma xenograft model. Hu5F9-G4 also blocked the binding of SIRPα on the surface of lymphoma cells to CD47 on the surface of macrophages, thus attenuating the “don't eat me” signals within this cancer cell type. Compared to treatment with IgG control antibody, a monoclonal antibody against CD47, B6H12, enhanced the *in vitro* phagocytotic activity of human macrophages against cancer cells and prolonged the survival of mice with intraperitoneal metastatic cancer (56). Macrophage-mediated phagocytosis of liver cancer cells can be enhanced by treatment with an anti-CD47 antibody, a SIRPα blocking antibody, or by blocking the CD47-TSP-1 interaction (64, 65). Attenuation of CD47-SIRPα signaling in cholangiocarcinoma promotes the phagocytotic potential of a variety of macrophage subpopulations and inhibits cholangiocarcinoma growth and intrahepatic metastasis (66). Anti-SIRPα antibody treatment leads to enhanced macrophage phagocytic activity (67) and reduced tumor progression in a mouse model of colon cancer (67) and CD47-SIRPα signaling promotes the expansion and metastasis of colon cancer cells in tumor microenvironments that are rich in tumor-associated macrophages (68). Two xenograft models of leiomyosarcoma in mice (via LMS04 and LMS05 tumor cell transplant) have also been treated with a humanized anti-CD47 monoclonal antibody, which increases the levels of macrophage-mediated phagocytosis of leiomyosarcoma tumor cells and inhibits the growth of primary tumors and the formation of lung metastases after primary tumor graft resection (30). Ring et al. (19) incubated different colorectal adenocarcinoma cell lines with human macrophages after treatment with an anti-SIRPα antibody (KWAR23) in combination with cetuximab or panitumumab (two types of treatments targeting epidermal growth factor receptor); these authors found that KWAR23 alone enhances macrophage-mediated phagocytosis of DLD-1 colorectal adenocarcinoma cells, and that the combination of KWAR23 and cetuximab increases the macrophage-mediated phagocytosis of DLD-1, LS, 174T, HT-29, and HCT 116 colon adenocarcinoma cells. Notably, the effectiveness of KWAR23 in inducing macrophage-mediated tumor cell phagocytosis was dependent upon the concentration of the antibody used, suggesting that the dose of CD47-SIRPα-targeting antibodies should be carefully optimized during the development of novel treatments that aim to inhibit CD47-SIRPα signaling (19). In this regard, future studies should aim to generate sufficient yields of CD47 inhibitors with a view to clinical use. It should also be noted that phagocytosis is regulated by the balance of pro-phagocytotic and anti-phagocytic signals, so the net effect of pro-phagocytotic signaling and phagocytosis antagonism will impact upon macrophage phagocytosis (69).

#### Impact of CD47/SIRPα Targeting on Macrophage Recruitment and Polarization

As well as increasing the level of phagocytosis, it is possible that blocking CD47 increases macrophage recruitment to tumors. For example, phagocytosis following anti-CD47 treatment can cause the secretion of chemokines and cytokines that recruit additional immune cells to tumors; these factors secreted in response to CD47-blocking therapies include monocyte chemotactic protein 3 (41). The CD47-blocking antibody, Hu5F9-G4, inhibits the growth of SCLC tumors *in vivo* and stimulates the release of chemokines that promote macrophage recruitment and activation, thus contributing to the efficacy of CD47-blocking therapy (41). Macrophage polarization state may also be altered by anti-CD47 therapy and one study of glioblastoma found that CD47 blockade converts tumor-associated macrophages into an anti-tumor state and increases macrophage recruitment into the tumor (70).

### Impact of CD47/SIRPα Targeting on the Adaptive Immune Response

CD47 blockade can promote the adaptive immune response, e.g., when treatment with an anti-CD47 antibody induced antigen-specific CD8^+^ T-cell proliferation and macrophage phagocytosis but reduced regulatory T-cell number in a colon cancer model, suggesting that anti-CD47 treatments can facilitate adaptive T-cell immune response (71). Similarly, a study by Liu et al. found that anti-CD47 antibody treatment inhibits tumor progression by enhancing the antigen-specific CD8^+^ T-cell response through dendritic cell-mediated presentation of tumor antigens to T-cells (72). In their study, Liu et al. also found using immunocompetent mouse models of lymphoma and lung cancer, that the anti-tumor responses to anti-CD47 treatment were partially dependent on an intact immune system (72). Furthermore, a separate study confirmed that anti-CD47 antibodies exert tumor-killing effects through the activation of CD8^+^ T-cells and dendritic cells (73), which phagocytose tumor cells and process specific antigens that lead to presentation of tumor cells to CD8^+^ T-cells, thereby activating tumor cell-specific adaptive immunity (73). Soto-Pantoja et al. have also shown that CD47 blockade induces a cytotoxic T-cell-dependent anti-tumor immune response in fibrosarcoma and that CD47 deletion in CD8^+^ T cells increases their anti-tumor activity, while raised CD47 expression was found to be associated with reduced infiltration of CD8^+^ T-cells in melanoma (74). In a study by Golubovskaya et al. (75), CD47-CAR-T cells that bind to CD47 were effective in killing pancreatic cancer cells. In addition, injection of CD47-CAR-T cells into immunodeficient mice blocked the growth of BxPC3 pancreatic xenograft tumors (75). Therefore, anti-tumorigenic immune cells directed to target CD47 could be used to inhibit tumorigenesis (76, 77). In another study, Zhou et al. (78) constructed tumor-activatable oxaliplatin prodrug vesicles, MPV-HOAD, and found that MPV-HOAD induced immunogenic cell death when combined with anti-CD47 antibody treatment and enhanced tumor immunogenicity; MPV-HOAD also induced the maturation of antigen presenting cells, thereby activating an anti-tumor immune response and effectively inhibiting the growth of both primary and abscopal tumors. In addition, after treatment with MPV-HOAD and CD47 blockade, CT26 colorectal tumor-bearing mice exhibited reduced tumor recurrence.

### Impact of CD47/SIRPα Targeting on Cell-Mediated Cytotoxicity

Anti-CD47 treatments can activate the antibody-dependent cellular cytotoxicity-mediated innate immune response. For instance, a study of head-and-neck squamous cell carcinoma demonstrated that raised CD47 expression is associated with decreased natural NK cell-mediated cytotoxicity, while anti-CD47 antibody treatment enhanced NK cell-mediated cytotoxicity (79). In particular, anti-CD47 antibodies can cause NK cell-mediated antibody-dependent cellular cytotoxicity in an Fc receptor-dependent manner (42), while anti-CD47 antibodies or fusion proteins can also function through the Fc active domain leading to antibody-dependent cell-mediated cytotoxicity (80). Feliz-Mosquea et al. (81) performed a quantitative high-throughput screening of anti-cancer drugs and administered an anti-CD47 antibody to a mouse 4T1 breast cancer model and concluded that blocking CD47: (i) enhances anthracycline-mediated tumor cytotoxicity; (ii) enhances macrophage-mediated cytotoxicity of breast cancer cells; and (iii) prevents anthracycline-mediated cardiomyocyte and tissue toxicity. A separate study showed that CD47/SIRPα blockade augments neutrophil-mediated antibody-dependent cytotoxicity (82). Other studies have identified direct cytotoxicity of CD47-targeting therapies on CD47^+^ cancer cells (83–85), but therapies targeting the SIRPα binding epitope do not cause direct cytotoxicity on tumor cells (41, 63).

### Impact of CD47/SIRPα Targeting on Tumor Cell Apoptosis

CD47 inhibition can also induce apoptosis of tumor cells; for instance, anti-CD47 antibodies increase tumor cell clearance by inducing apoptosis through a caspase-independent mechanism (71). Researchers have treated mice with KPMM2 cells, which are human myeloma cells transplanted with a mouse monoclonal single-chain antibody fragment targeting CD47 (84). Treatment with the KPMM2 cells induced cancer cell apoptosis and can be combined with other chemotherapies (83), thus providing a putative treatment for MM that may be used in combination with conventional chemotherapy (86). CD47 blockade using an antibody that is specific for an extracellular domain of CD47 augmented apoptosis of leukemia and myeloma cells (83, 84). Song et al. (87) transfected a CD47-siRNA lentiviral vector into the KG1a AML cell line to inhibit the expression of CD47 and found that this treatment reduced the expression of anti-apoptotic genes including Bcl-2, Bcl-xl and MCL-1. Pietsch et al. (88) assessed the anti-leukemia activity of several anti-CD47 antibodies in mice and cynomolgus monkeys; after transplantation of HL60, MV4-11, and Kasumi-3 AML cells, animals were treated with the Fc region of various anti-CD47 monoclonal antibodies (including C47B157, C47B161, C47B222, and B6H12.2) that led to reductions in the levels of peripheral AML cells. Interestingly, treatment of human or murine acute lymphoblastic leukemia (ALL) T-cells with a soluble CD47 agonist peptide induces changes in reactive oxygen species (ROS) production (89) and caspase-independent and calcium-dependent leukemia cell death; this study also showed that PKHB1-treated cells could be used as a prophylactic to prevent tumorigenesis (90). Leclair et al. (91) compared the Jurkat T-cell line (cells that expresses high levels of CD47) with the JC47-2-4 cell line (cells that expresses low levels of CD47). It was found that the anti-CD47 antibody, CC2C6, induces Jurkat cell death in a CD47-dependent manner. In addition to directly inducing cell death via blockade of CD47-SIRPα signaling, this study showed that CC2C6 synergizes with low-dose chemotherapeutic agents that induce classical apoptosis, resulting in an effective combination of treatments without the need for high-dose chemotherapy, which is often associated with long-term side-effects in pediatric ALL patients (91). A recent study has also shown that treatment of transgenic mice with the hyaluronan synthesis inhibitor, 4-methylumbelliferone (4MU), induces apoptosis and reduces inflammation, steatosis, and the expression of cancer stem cells markers (92). In the presence of cancer stem cells, 4MU downregulates the expression of CD47 on HCC cells and promotes phagocytosis of antigen presenting cells; in combination with adenovirus encoding interleukin-12 genes (AdIL-12), 4MU elicits a potent cytotoxic T-cell-specific response and prolongs survival in a hepatocellular carcinoma model established in fibrotic liver (93).

### Impact of CD47/SIRPα Targeting on Tumor Cell Proliferation and Migration

Anti-CD47 therapies may inhibit tumor cell proliferation, given that CD47 promotes tumor cell proliferation and metastasis (94). In a mouse model of lymphoma, anti-CD47 antibodies prevent lymphoma cell proliferation and prolong survival (95). Treatment with a CD47-blocking antibody also leads to delayed progression of metastasis and prolonged survival in mice with pancreatic tumors (6, 96). CD47 binding with TSP-1 inhibits nitric oxide (NO) signaling and limits NO production, which accelerates osteoclast formation and activation, and in turn promotes tumor cell metastasis to bone (97). Meanwhile, blocking the interaction between CD47 and TSP-1 with anti-TSP-1 antibodies disrupts tumor-induced osteoclast formation (98), but deficiency of CD47 alone does not fully recapitulate the effects of antibody-mediated blockade of TSP-1 in either murine or human osteoclasts cells (12). Kim et al. (33) constructed a mouse model of myeloma cell transplantation and treated these mouse with B6H12, showing that B6H12 inhibits myeloma cell growth in the bone marrow microenvironment. Li et al. (53) showed that when a shCD47 lentiviral vector was used to reduce the expression of CD47 on glioma stem cells, the growth potential and differentiation of these cells were inhibited. Li et al. (53) also showed in immunocompetent mouse glioma models that blocking CD47 inhibits tumor growth and prolongs survival. Furthermore, synergistic activities of anti-CD47 therapies and chemotherapeutic drugs can enhance the efficacy of chemotherapy in patient-derived HCC xenograft mouse models and inhibit the growth and metastasis of liver cancer cells *in vivo* (99, 100). Using an antibody-free approach, the NSCLC cell lines, A549 and NCI-H520, have been transfected with siRNA against CD47 to reduce the endogenous expression of CD47 (101). Reduction of CD47 expression by siRNA treatment inhibited the migration and invasion of A549 and NCI-H520 cells on a microfluidic chip (102). Furthermore, CD47 overexpression in A549 and NCI-H520 cells by transfection with pcDNA3.1-3xFlag-CD47 conversely enhanced the migration and invasion potentials of A549 and NCI-H520 cells (5). These *in vitro* findings were confirmed *in vivo* using a mouse xenograft model of NSCLC (103). Given that NSCLC cell viability did not decrease following siRNA transfection to reduce CD47 expression, it appears that the treatment-dependent reductions in cell migration and invasion can occur without cytotoxicity. Xu et al. (104) treated a mouse osteosarcoma cell line (LM8) and a human osteosarcoma cell line (KRIB) with an anti-CD47 antibody (B6H12) or control IgG antibody; their study showed that in a mouse model of metastatic KRIB osteosarcoma, there is a positive correlation between CD47 expression in the KRIB cells and the extent of metastasis, and following treatment with the anti-CD47 antibody, osteosarcoma cells were less invasive than IgG-treated cells. By treating these mice with an anti-CD47 antibody (B6H12), spontaneous metastasis of xenograft osteosarcoma cancer cells to the lung was inhibited, and phagocytosis of the osteosarcoma cells by macrophages enhanced (104). Cell division control protein 42 (Cdc42), a member of the Rho family of small GTPases, has been identified as a regulator of metastasis and is activated downstream of CD47 to promote cell formation (105, 106). Cdc42 expression level is positively correlated with CD47 expression level in A549 and NCI-H520 lung cancer cell lines (107). In A549 and NCI-H520 lung cancer cells that ectopically express CD47, decreasing Cdc42 levels reduced cancer cell migration and invasion (108). Conversely, when the expression of Cdc42 is increased, the migration and invasion ability of lung tumor cells is enhanced (109). In 80 patients with advanced NSCLC, the expression levels of CD47 and Cdc42 were shown by immunohistochemistry to be positively correlated (5). If CD47 controls Cdc42 expression in NSCLC cells, and Cdc42 in turn mediates the invasive phenotype of these cancer cells, then blocking CD47 or inhibiting Cdc42 expression may become a new method of inhibiting the metastasis of NSCLC cells.

## Anti-CD47 Therapies in Clinical Development and Their Tumor Specificity

While the CD47 signaling cascade remains incompletely understood, recent studies have improved understanding of CD47-dependent signaling and given rise to the development of CD47-targeting anti-cancer therapies that inhibit primary tumor growth and reduce metastasis in various types of cancer. Studies using an anti-CD47 antibody (IgG1 C47B222-CHO) in primary AML patients, however, showed that this treatment only affected the number of AML cells in the spleen without affecting the overall burden of myeloid leukemia (88). Nevertheless, several CD47-targeting antibodies or drugs have reached clinical trials (Table 1), including Hu5F9-G4 (Forty-Seven), CC-90002 (Celgene), TTI-621 (Trillium), ALX148 (Alexo Therapeutics), SRF231 (Surface Oncology), SHR-1603 (Hengrui), and IBI188 (Innovent Biologics). Among these, Hu5F9-G4, CC-90002, and IBI188 are anti-CD47 antibodies, while TTI-621 and ALX148 are SIRPα-Fc fusion proteins (18, 110). Advani et al. (111) recruited 22 patients with lymphoma (15 with DLBCL, 7 with follicular lymphoma, and 95% of whom had rituximab refractory disease) to carry out a phase 1b clinical trial; in patients with invasive and indolent lymphoma, Hu5F9-G4-associated phagocytosis was enhanced by the addition of rituximab; furthermore, adverse reactions experienced by the treated patients (e.g., anemia, nausea, diarrhea, and infusion-related reactions) occurred only in the first few weeks and were not followed by a significant increase in clinically safety events in the later stages of the trial. Recent studies have also proposed another method for reducing CD47 expression and decreasing tumor cell growth by using miRNA to inhibit the expression of CD47 (112, 113). Given that there is a negative correlation between miR-708 and CD47 in T-ALL patients (114), the recovery of miR-708 expression could regulate the expression of CD47. Furthermore, a combination of miR-708 and anti-CD47 antibody treatment increases the phagocytic activity of macrophages against leukemic CEM cells compared with either agent alone, and could therefore be used as a combinatorial anti-leukemia therapy in future studies (114).

**Table 1:** The development of anti-CD47 therapies.

**Name**	**Target**	**Progress**	**Company**
Hu5F9-G4	CD47	Phase I/II clinical	Stanford University Forty Seven
TI-061	CD47	Phase I/II clinical	Arch Oncology
TTI-622	SIRPα	Phase I clinical	Trillum Therapeutics
TTI-621	SIRPα	Phase I clinical	Trillum Therapeutics
SRF231	CD47	Phase I clinical	Surface Oncology
SHR-1603	CD47	Phase I clinical	Hengrui
OSE-172	SIRPα	Phase I clinical	Boehringer Ingelheim OSE Immunotherapeutics
NI-1701	CD47 CD19	Phase I clinical	Novimmune TG Therapeutics
IBI188	CD47	Phase I clinical	Innovent Biologics
CC-95251	SIRPα	Phase I clinical	Celgene
CC-90002	CD47	Phase I clinical	Celgene Inibrx
AO-176	CD47	Phase I clinical	Arch Oncology
ALX148	SIRPα	Phase I clinical	ALX Oncology
IMM01	SIRPα	Applying for a clinical trial	ImmuneOnco Biopharma
TJC4	CD47	Applying for a clinical trial	I-MAB Biopharma
TJC4-CK	CD47	Preclinical trial	I-MAB Biopharma
SY102	CD47	Preclinical trial	Saiyuan
SL-172154	CD40L SIRPα	Preclinical trial	Shattuck Labs
PSTx-23	CD47	Preclinical trial	Paradigm Shift Therapeutics
PDL1/CD47BsAb	PD-L1 CD47	Preclinical trial	Hanmi Pharmaceuticals
NI-1801	CD47 MSLN	Preclinical trial	Novimmune
MBT-001	CD47	Preclinical trial	Morphiex
LYN00301	CD47	Preclinical trial	LynkCell
IMM2504	PDL1 CD47 VEGF	Preclinical trial	ImmuneOnco Biopharma
IMM2502	PDL1 CD47	Preclinical trial	ImmuneOnco Biopharma
IMM03	CD20 CD47	Preclinical trial	ImmuneOnco Biopharma
IMM02	SIRPα VEGFR1	Preclinical trial	ImmuneOnco Biopharma
IMC-002	CD47	Preclinical trial	ImmuneOncia Therapeutics
IBI322	PDL1 CD47	Preclinical trial	Innovent Biologics
HMBD-004B	CD47	Preclinical trial	Hummingbird Bioscience
HMBD-004A	CD47 CD33	Preclinical trial	Hummingbird Bioscience
HLX24	CD47	Preclinical trial	Henlius
FSI-189	SIRPα	Preclinical trial	Forty Seven
DSP107	SIRPα 4-1BBL	Preclinical trial	KAHR medical
CTX-5861	SIRPα	Preclinical trial	Compass Therapeutics
BAT6004	CD47	Preclinical trial	Bio-Thera
AUR-105	CD47	Preclinical trial	Aurigene
AUR-104	CD47	Preclinical trial	Aurigene
ANTI-CD47 MAB	CD47	Preclinical trial	Biocad
ABP-500	CD47 TWEAKR	Preclinical trial	Abpro
ABP-160	PDL1-CD47	Preclinical trial	Abpro
BH-29xx	PD1-CD47	Preclinical trial	Beijing Hanmi

Despite encouraging findings from studies of the impact of anti-CD47 therapy on cancer progression, the effectiveness of anti-CD47 treatments can vary between cancer types, different studies, and even different cancer types (Table 2); in other words, the therapeutic efficacy of anti-CD47 treatments may be tumor-specific. The different responses between tumor types could be a result of differences in: (i) drug delivery method (route, vehicle, frequency, efficiency); (ii) drug compartmentalization in the tumor cell; (iii) stage of cancer progression; (iv) capability of the immune system; and (v) acquired drug resistance. Potential factors contributing to drug resistance include (but are not limited to) tumor heterogeneity, changes in the tumor microenvironment, drug inactivation, decreased drug absorption or increased drug release from the tumor cells, activation of tumor cell survival pathways, and epigenetic changes (121, 122). In preclinical studies, the variation in response to anti-CD47 treatment between cancer type and individual studies could also be a result of differences in the type of tumor model(s) used to test treatment efficacy. For example, many of the studies that have been published to date make use of xenograft tumor models, while fewer studies have instead employed syngeneic tumor models; future studies should aim to develop and utilize tumor models that are as consistent as possible with the pathology of human tumors. The strengths and weaknesses of preclinical cancer models have been thoroughly reviewed elsewhere (123). While no single tumor model can fully recapitulate the pathogenesis and progression of malignancy in humans, consistent findings from a wide variety of different types of mouse models could ultimately lead to the development of novel CD47-targeting treatments for cancer patients.

**Table 2:** Examples of the impact of anti-CD47 treatments in different types of cancer.

**Cancer**	**Treatment**	**Target**	**Impact**	**References**
Acute myeloid leukemia (AML)	B6H12	CD47	Increased phagocytotic activity of macrophages; reduced tumor cell engraftment	(38)
	C47B157, C47B161, C47B222, B6H12.2	CD47	Reduced peripheral AML cell number	(88)
	IgG1 C47B222-CHO	CD47	Reduced AML cell number in the spleen but not bone marrow	
	ZF1	CD47	Increased phagocytosis of leukemia stem cells	(59)
Acute lymphoblastic leukemia (ALL)	CC2C6	CD47	Increased Jurkat cell death	(91)
Lymphoma	Rituximab and Hu5F9-G4	CD47	Increased cancer cell phagocytosis	(111)
Multiple myeloma (MM)	KPMM2 cells	CD47	Increased cancer cell apoptosis; increased survival	(84)
	Anti-TSP-1 antibody	TSP-1	Reduced osteoclast formation	(12)
	B6H12	CD47	Increased phagocytosis of myeloma cells; reduced tumor growth; increased survival	(33)
Liver cancer	Anti-CD47 antibody	CD47	Increased phagocytotic activity of macrophages; reduced tumor growth	(65)
	4MU	CD47	Reduced CD47 expression on hepatic cancer cells	(115)
	4MU and AdIL-12	CD47	Increased cytotoxic T-cell-specific response	
Colorectal cancer	Hu5F9-G4	CD47	Unaltered phagocytosis	(116)
	KWAR23 and cetuximab	SIRPα	Increased macrophage-mediated phagocytosis of colorectal adenocarcinoma cells; reduced tumor growth	(19)
Pancreatic cancer	Anti-hCD47 (B6H12)	CD47	Unaltered tumor growth	(6)
	Hu5F9-G4	CD47	Unaltered phagocytosis	(116)
	CD47-blocking antibody	CD47	Reduced metastasis and increased survival	(6)
	CD47-CAR-T cells	CD47	Reduced pancreatic cancer cell number; reduced tumor growth	(75)
	Nanoparticles loaded with gemcitabine and anti-CD47 antibody	CD47	Increased pancreatic cancer cell apoptosis; reduced tumor growth	(117)
	Mithomycin A-loaded nanoparticles	CD47	Reduced CD47 expression in tumor xenografts	(118)
Leiomyosarcoma	A humanized anti-CD47 monoclonal antibody	CD47	Increased phagocytosis and reduced tumor growth and metastasis	(30)
Breast cancer	Anthracyclines and Anti-CD47 antibody	CD47	Reduced invasive breast tumor growth	(81)
	Hu5F9-G4	CD47	Unaltered phagocytosis	(116)
Small cell lung cancer (SCLC)	Hu5F9-G4	CD47	Unaltered phagocytosis	(116)
	Hu5F9-G4	CD47	Reduced SCLC tumor growth	(41)
Non-small cell lung cancer (NSCLC)	siRNA against CD47	CD47	Reduced CD47 expression	(101)
Urinary and bladder cancer	Anti-CD47 single-chain antibody nanoparticles	CD47	Reduced tumor cell survival	(119)
Glioblastoma	shCD47 lentiviral vector	CD47	Reduced CD47 expression	(53)
	MIAP301	CD47	Reduced tumor growth and increased survival	
	Anti-CD47 monoclonal antibody	CD47	Increased formation of autophagosomes	(120)
Osteosarcoma	Hu5F9-G4	CD47	Unaltered phagocytosis	(116)
	B6H12	CD47	Reduced metastasis	(104)

## Biosafety Problems and Future Perspectives

**Figure 3:**
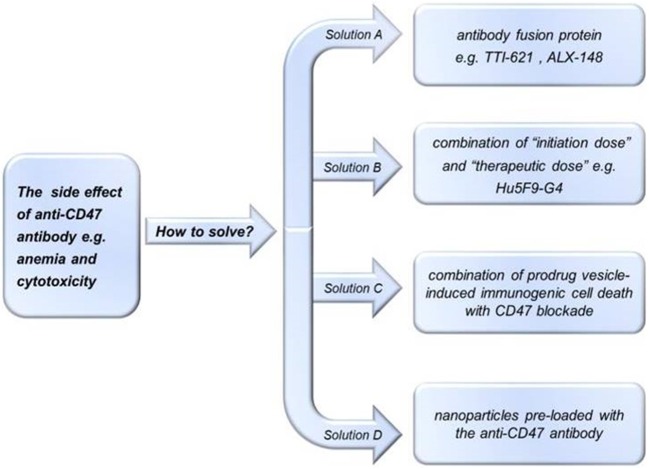
Potential methods of avoiding the side effects of anti-CD47 treatments. To reduce side effects of treatment with anti-CD47 antibodies, investigators could use: (A) antibody fusion proteins e.g., TTI-621 (133) or ALX-148 (134); (B) “initiation doses” followed by “therapeutic doses” e.g., Hu5F9-G4 (111); (C) pro-drug vesicle-induced immunogenic cell death combined with CD47 blockade (78); or (D) tumor-targeting nanoparticles loaded with anti-CD47 antibody (135).

Preclinical studies using anti-CD47 antibodies in mice and macaques suggested that these therapeutics are well-tolerated (33, 62, 124). In 2017, however, Arch Oncology discontinued a phase I/II clinical trial of an anti-CD47 monoclonal antibody, Ti-061, while in 2018, Celgene discontinued a clinical trial of the anti-CD47 monoclonal antibody, CC-90002 for the treatment of AML. Given that CD47 is ubiquitously expressed, potential problems with using anti-CD47 antibodies as anti-cancer treatments include possible off-target effects such as anemia (73). For instance, CD47 is expressed by non-malignant cells of the hematopoietic system (28), including normal red blood cells, senescent red blood cells, and platelets (125, 126). Buatois et al. (127) showed that Hu47F9-G4 alone or in combination with other antibodies may cause accidental killing of normal red blood cells, potentially resulting in anemia. To alleviate this side-effect, one study (111) proposed to give short-term low-dose Hu5F9-G4 in combination with rituximab, which causes predictable and transient mild anemia due to selective elimination of aged red blood cells, followed by treatment with compensatory reticulocytes (127). The toxicity of anti-CD47 antibodies, however, appears to be Fc-dependent, given that anti-CD47 antibodies and SIRPα-Fc fusion proteins give rise to this toxicity, while high-affinity SIRPα monomers do not (33, 62, 124). These findings suggest that future studies should aim to optimize the structure of the anti-CD47 therapeutic when attempting to design novel drugs without unwanted side-effects. For example, next-generation SIRPα-Fc fusion protein variants have been generated to enhance their binding to CD47; these higher-affinity SIRPα variants bind to CD47 with greater potency compared to wild-type SIRPα (124), and were effective against hematologic and solid tumors in preclinical studies, but have not yet progressed to clinical trials (124, 128). In another recent study, Sim et al. describe their discovery of high affinity, pan-allelic, and pan-mammalian antibodies against SIRPα (129). Prior to this, Ho et al. described the production of high affinity CD47 ectodomain as an antagonist of SIRPα to increase antibody-dependent phagocytosis (130). As an aside to this, patient age should be considered in future studies of CD47/SIRPα-targeted therapies, given that older erythrocytes may be more susceptible to phagocytosis (131, 132). How to reduce or avoid damage to normal cells while exerting anti-tumor effects is one of the problems that needs to be considered when designing anti-CD47 therapies (Figure 3) (81). TTI-621 (Trilium) is an example of an antibody fusion protein consisting of the N-terminal V domain of human SIRPα fused to the human IgG1 Fc region that was developed to avoid damage to normal cells while still being able to increase the removal of tumor cells; although TTI-621 resulted in anemia and cytopenia in primates, healthy human erythrocytes showed minimal binding activity to TTI-621, enabling the therapy to be administered at low therapeutic doses whilst still maintaining sufficient receptor binding (133). The molecular structures and treatment regimens of ALX148 (134) and Hu5F9-G4 have also been designed with a view to reducing cytotoxicity.

The so-called “antigen sink” effect could also pose a problem in the development of anti-CD47 treatments. The ubiquitous expression of CD47 means that large initiation doses and/or frequent administrations may be required for a drug to achieve an effective therapeutic blockade of CD47. In a phase I trial of Hu5F9-G4, for example, an “initiation dose” was followed by a “therapeutic dose” (111). Alternatively, SIRPα has a more restricted histological distribution vs. CD47, which could lead to less toxicity and greater blockade when therapeutically targeted (128). SIRPα is highly expressed, however, on myeloid cells and central and peripheral nervous system cells (136), so the potential for neurological side-effects should be considered when using therapeutics that target SIRPα. Furthermore, cross-reactivity may occur between other SIRP family members (SIRPβ and SIRPγ) as a result of their sequence similarity, while that at least 10 polymorphisms of human SIRPα have been identified (137). The consequences of targeting these different isoforms of receptors is not yet clear (20, 137). In future studies, methods of targeting CD47 and its ligands specifically on tumor cells should be investigated; these could include novel drug delivery vehicles such as modified biomimetic nanoparticles (135) or quorum-sensing bacteria (138). For example, a recent study showed that a bio-responsive fibrin gel solution containing anti-CD47-conjugated nanoparticles modulate the immune response and induce the phagocytosis of tumor cells by blocking CD47; furthermore, this treatment resulted in an augmented T cell-mediated immune response and activation of tumor-associated macrophages (135). In another recent study by Chowdhury and colleagues, quorum-sensing bacteria were used to deliver a single-chain antibody fragment that targets CD47 and showed that this strategy leads to systemic anti-tumor immunity and reduced tumor progression (138). Multi-functional iron oxide magnetic nanoparticles have been developed as vehicles for selective treatment of pancreatic cancer, including for the simultaneous delivery of gemcitabine and anti-CD47 antibodies (117). The therapies delivered via nanoparticles did not have additional cytotoxicity and the nanoparticle-mediated delivery of the anti-CD47 antibody to tumor cells caused greater PDX models of PDAC apoptosis compared with free antibody in the treatment of pancreatic cancer (117). Mithomycin A-loaded nanoparticles can also be used to down-regulate the expression of CD47 and to increase therapeutic efficacy in BxPC-3 tumor xenografts in athymic mice (118). Furthermore, Davis et al. (139) combined anti-CD47 antibodies with SERS nanoparticles and showed by applied Raman spectroscopy that the antibody-loaded nanoparticles targeted ovarian cancer cells. Nanoparticle delivery methods could be further optimized in future studies, for example to prevent the circulating nanoparticles from being cleared by the reticuloendothelial system. If nanomedical technologies continue to advance at the current rate, it is possible that drug-loaded nanoparticles targeting CD47 will ultimately be developed for the treatment of CD47-overexpressing tumors. As well as new delivery methods, it is also possible that intra-tumoral drug administrations may lead to greater drug delivery and efficacy and reduced toxicity compared with intra-peritoneal or sub-cutaneous delivery routes.

As mentioned above, non-optimal tumor responses following anti-CD47 treatment have led to studies investigating the use of combination therapies including tumor cell-specific opsonizing antibodies and T-cell checkpoint inhibitors. In a mouse model of non-Hodgkin lymphoma, for example, an anti-CD47 antibody (BRIC126 or B6H12) combined with a clinically-approved anti-CD20 antibody (rituximab) resulted in human lymphoma cell ablation in xenograft models of tumor progression (42, 140). Such approaches can be used in attempts to achieve increased tumor specificity and decreased on-target toxicity to CD47-expressing non-malignant cells. In a similar approach, another bispecific antibody that targets CD47 and CD19 (NI-1701) was designed for B-cell lymphoma and refractory leukemia (127), while a fusion protein targeting CD47 and PD-L1 has also been shown to have anti-tumor efficacy by activating the adaptive immune response (141, 142). The phagocytotic and anti-tumor effects of high-affinity SIRPα proteins that block CD47 are also enhanced when combined with tumor-opsonizing antibodies including rituximab, trastuzumab, or cetuximab (124), or the anti-CD56 antibody, lorvotuzumab (41). Anti-SIRPα antagonists have also been combined with tumor-opsonizing antibodies such as rituximab to show anti-tumor efficacy *in vitro* (130) and in xenograft lymphoma and syngeneic colon cancer models (143), while bispecific agents have been produced whereby the binding domain of SIRPα is fused to a tumor-targeting antibody such as anti-CD33 (144). While clinical trials have begun to assess CD47-blocking agents in combination with rituximab, cetuximab, and trastuzumab (128), it is also possible that future improvements in cancer screening and precision medicine will enable the identification and stratification of specific tumor types and/or stages of cancer that would be most amenable to treatment with a certain type or types of anti-CD47 treatment.

## Conclusions

Given that CD47-SIRPα signaling enables malignant cells to avoid macrophage-mediated phagocytosis, the inhibition of the CD47-SIRPα signaling axis represents a promising therapeutic strategy for the treatment of cancer, and multiple CD47 targeted drugs have entered the clinical trials. However, there are a series of biosafety problems with such treatments including anemia, and preclinical studies have demonstrated different effects of CD47-targeted drugs on different tumor types. In addition, the signaling mechanisms that are upstream and downstream of the CD47-SIRPα complex are incompletely understood. A better understanding of the mechanisms that allow tumor cells to avoid immune clearance along with improvements in the delivery of anti-CD47 agents could lead to the development of novel and effective anti-cancer treatments that enhance the phagocytosis of malignant cells.

## Author Contributions

WZ, QH, and WX did the drafting. YZ, JP, HX, HZ, and JX were responsible for looking up and collecting information. CE and HJ were responsible for revising the manuscript.

### Conflict of Interest

The authors declare that the research was conducted in the absence of any commercial or financial relationships that could be construed as a potential conflict of interest.
